# Price Is a Feature of Person-Centered Abortion Care

**DOI:** 10.2196/112216

**Published:** 2026-10-13

**Authors:** Tracy A Weitz, Angel M Foster

**Affiliations:** 1 Department of Sociology, American University, 4400 Massachusetts Ave NW, Watkins Building, G10A, Washington, DC, 20016, United States, 202-885-2475; 2 Faculty of Health Sciences, University of Ottawa, Ottawa, ON, Canada

**Keywords:** abortion, telemedicine, price, patient-centered care, affordability

## Abstract

Schroeder and colleagues contribute to the understanding of person-centered abortion care by examining preferences for telemedicine and facility-based medication abortion care. We argue that because cost was very or extremely important to three-quarters of participants, price should itself be treated as a feature of care, and studies of patient preferences should attend to the actual prices patients face across care options.

## Introduction

Schroeder and colleagues [1] make an important contribution to the study of abortion preferences by expanding the question beyond how an abortion is clinically performed (medications or instruments) to how medication abortion care is delivered: through telemedicine or at a brick-and-mortar facility. Their focus on person-centered abortion care is particularly valuable as models of abortion provision evolve. Yet their analysis also illustrates an important limitation in how abortion researchers conceptualize patient preferences: the tendency to locate the meaningful attributes of care primarily at the level of service delivery while giving less attention to the economic conditions that determine which forms of care patients can actually obtain.

## Cost Is Important Even When It Does Not Distinguish Care Models

One of the most striking findings made by Schroeder and colleagues [1] receives little attention in their conclusions. Cost was “extremely important” to 56.9% of participants and “very important” to another 19.6%. Thus, more than three-quarters (76.5%) considered cost very or extremely important. Cost did not significantly differ in importance between participants receiving telemedicine and in-person care. But a lack of difference between groups should not be interpreted as a lack of importance to patients. Indeed, ultrasound was extremely important to only 25.9% of participants overall yet receives considerable attention from the authors because its importance differed significantly by care model. The study design identifies characteristics that distinguish telemedicine from in-person patients; it does not establish that characteristics that fail to distinguish the groups are unimportant to abortion seekers.

## Cost Is Experienced Differently Across Payment Sources

Interpreting the cost finding is further complicated because “cost” does not represent the same experience for all participants. Among study participants, 55.4% paid with their own or someone else’s money, 9.6% received assistance from a clinic or abortion fund, 21.0% used Medicaid, and 11.5% used private insurance or another source. During the study period, 4 of the 6 study states permitted state Medicaid funding of abortion [2]. For a patient whose abortion is fully covered by Medicaid, the importance of the “cost” of abortion has a different meaning than for someone required to pay the full price out of pocket who would otherwise qualify for Medicaid coverage of abortion care if it were not excluded by their state. Yet the analysis does not stratify the importance of cost by payment source, state Medicaid policy, or economic vulnerability.

## Price Shapes the Choices Available to Patients

This distinction matters because decades of research demonstrates that abortion prices affect whether and when women and other pregnancy-capable people obtain abortions [3]. When Medicaid covers abortion, use increases; when coverage is unavailable, some Medicaid-eligible people are unable to obtain a wanted abortion. Those who do obtain care often divert money from rent, utilities, food, and other necessities to pay for it. For a population disproportionately living on low incomes, abortion care paid for out of pocket can constitute a catastrophic health expenditure. Cost is therefore not simply another preference alongside privacy, ultrasound, convenience, or clinician interaction. It can constrain the set of options from which a patient is able to choose.

The actual prices of the care models investigated by Schroeder and colleagues [1] are therefore important for interpreting the findings, yet they are not reported. Were participants choosing between telemedicine and in-person services that cost the same amount? Did prices vary across the 4 participating organizations? These questions matter because preferences are expressed within the alternatives patients encounter. If telemedicine and in-person care were priced the same, why would a patient not prefer an in-person visit if it included an ultrasound at no additional expense? Likewise, the value of avoiding a clinic visit may look different when doing so also reduces the price of care.

The separation of price from abortion method and delivery preferences has a history. When mifepristone entered US abortion practice after the Food and Drug Administration’s approval in 2000, abortion providers commonly priced medication and instrumentation abortion care similarly [4], in part to avoid influencing patients’ method choice through price. Even though medication abortion subsequently required fewer visits, less testing, and no in-person follow-up, patient prices did not decline correspondingly [5]. The underlying principle was that patients should choose an abortion method based on their preferences rather than because providers had financially advantaged one method over another. But treating equal prices as a prerequisite for autonomous choice can obscure another form of inequity—the inability of some patients to afford care at all.

## Telemedicine Among Abortion Providers Protected by Shield Laws Has Changed the Economics of Abortion Care

The rapid expansion of abortion with medication obtained via telemedicine makes reconsideration of this assumption of the importance of price parity particularly important. Schroeder and colleagues [1] collected data from May 2021 through March 2023, before abortion shield law provision began, a limitation the authors appropriately acknowledge. This legal protection allows clinicians in a state to legally provide medication abortion to abortion seekers regardless of their state of residence. Since then, telemedicine from abortion providers protected by shield laws has transformed not only where abortion is provided but also its economics. Shield law–protected services have made medication abortion available for substantially less than the approximately $500 (US $) commonly charged for facility-based medication abortion care, with some shield law–protected services charging $150 or less and others using sliding scales that reach $5 [6]. The result has been dramatic. In March 2023, when the last patient was enrolled in the study by Schroeder and colleagues [1], only 9% of abortions in the formal US health care system were provided via telemedicine. In December 2025, almost 30% of all abortion provision was via telemedicine [7]. Research on shield law–protected medication abortion recipients and discrete-choice studies likewise suggest that price plays an important role in abortion-seekers’ decisions [8-10].

## Conclusion

Schroeder and colleagues [1] are right that person-centered abortion care requires maintaining different models of care because patients value different features. Their findings show meaningful variation in preferences for receiving care at home, meeting clinicians in person, and obtaining ultrasonography. But person-centered care cannot be defined only by whether patients prefer features of the care delivery process. Price itself is a feature of care. If abortion researchers seek to understand the choices patients make among evolving models of care, they must study not only which services patients prefer, but what those services cost them and which choices they can actually afford.
